# Quantitative Analysis of the Microtubule Interaction of Rabies Virus P3 Protein: Roles in Immune Evasion and Pathogenesis

**DOI:** 10.1038/srep33493

**Published:** 2016-09-21

**Authors:** Aaron Brice, Donna R. Whelan, Naoto Ito, Kenta Shimizu, Linda Wiltzer-Bach, Camden Y. Lo, Danielle Blondel, David A. Jans, Toby D. M. Bell, Gregory W. Moseley

**Affiliations:** 1 Viral Pathogenesis Laboratory, Department of Biochemistry and Molecular Biology, Bio21 Institute, The University of Melbourne, Melbourne, Victoria, 3010, Australia; 2 School of Chemistry, Monash University, Clayton, Victoria 3800, Australia; 3 Laboratory of Zoonotic Diseases, Faculty of Applied Biological Sciences, Gifu University, 1-1 Yanagido, Gifu 501-1193, Japan; 4 The United Graduate School of Veterinary Sciences, Gifu University, 1-1 Yanagido, Gifu 501-1193, Japan; 5 Nuclear Signaling Laboratory, Department of Biochemistry and Molecular Biology, Monash University, Clayton, Victoria 3800, Australia; 6 Viral Pathogenesis Laboratory, Department of Biochemistry and Molecular Biology, Monash University, Clayton, Victoria 3800, Australia; 7 Monash Micro Imaging, 27-31 Wright Street, Clayton, Victoria, 3168, Australia; 8 Unité de Virologie Moleculaire et Structurale, CNRS, UPR 3296, 91198 Gif sur Yvette Cedex, France

## Abstract

Although microtubules (MTs) are known to have important roles in intracellular transport of many viruses, a number of reports suggest that specific viral MT-associated proteins (MAPs) target MTs to subvert distinct MT-dependent cellular processes. The precise functional importance of these interactions and their roles in pathogenesis, however, remain largely unresolved. To assess the association with disease of the rabies virus (RABV) MAP, P3, we quantitatively compared the phenotypes of P3 from a pathogenic RABV strain, Nishigahara (Ni) and a non-pathogenic Ni-derivative strain, Ni-CE. Using confocal/live-cell imaging and *d*STORM super-resolution microscopy to quantify protein interactions with the MT network and with individual MT filaments, we found that the interaction by Ni-CE-P3 is significantly impaired compared with Ni-P3. This correlated with an impaired capacity to effect association of the transcription factor STAT1 with MTs and to antagonize interferon (IFN)/STAT1-dependent antiviral signaling. Importantly, we identified a single mutation in Ni-CE-P3 that is sufficient to inhibit MT-association and IFN-antagonist function of Ni-P3, and showed that this mutation alone attenuates the pathogenicity of RABV. These data provide evidence that the viral protein-MT interface has important roles in pathogenesis, suggesting that this interface could provide targets for vaccine/antiviral drug development.

The microtubule (MT) cytoskeleton is an extensive cytoplasmic network of filaments composed of α-/β-tubulin subunits, which has diverse functions in cellular processes including signal transduction, vesicular and organelle transport, and nucleocytoplasmic protein trafficking, many of which are regulated by a class of cellular proteins called MT-associated proteins (MAPs)^1^,^2^,^3^,^4^. Viruses commonly exploit MT-dependent cytoplasmic trafficking to deliver virions/genomes to sites of replication and egress^5^,^6^,^7^,^8^, but several reports suggest that viral interactions with MTs might also enable subversion of the biology of infected cells *via* discrete interactions formed by specific “viral MAPs”^9^. Among these is P3 protein of the lethal lyssavirus rabies virus (RABV), an isoform of the phosphoprotein (P protein) that is expressed in infected cells^10^.

P protein isoforms have been shown to have antagonistic function toward antiviral innate immune signaling by type-I interferon (IFN) cytokines, by inhibiting both IFN induction and IFN-dependent signaling^11^,^12^,^13^,^14^,^15^,^16^. The latter involves interaction of P protein with the transcription factors signal transducers and activators of transcription (STATs), that mediate intracellular signaling by IFNs, to inhibit their nuclear translocation, DNA interaction and consequent activation of IFN-stimulated gene expression^11^,^12^,^13^,^14^,^15^,^16^,^17^,^18^. Notably, P3 has been shown to interact with MTs to antagonize IFN signaling through a mechanism dependent on the integrity of the MT cytoskeleton, a property also observed in cells infected by RABV^19^. This appears to involve association of STAT1 with MTs *via* P3, which results in inhibition of STAT1 nuclear translocation^19^. However, in common with many other viral MAPs, the relationship of the P3-MT interaction with viral pathogenesis has not been directly examined, due primarily to the lack of specific inhibitory mutations with which the functional significance of the interaction can be interrogated using P3 protein or infectious virus.

To address this issue, we have analyzed the MT-association and IFN-antagonist function of P3 proteins derived from two strains of RABV, the pathogenic, IFN-resistant strain Nishigahara (Ni), and the non-pathogenic, IFN-sensitive derivative strain of Ni, Ni-CE^20^,^21^. Recent analysis of these strains identified the P protein-encoding *P* gene as a critical determinant of viral susceptibility to IFN and of pathogenicity in mice, by showing that replacement of the Ni-CE *P* gene with that of Ni (generating the recombinant CE-NiP virus) resulted in increased IFN resistance due to increased IFN-antagonistic function, and greater virulence following intracerebral inoculation^13^,^20^,^21^,^22^. Thus, this system provides a means to analyze potential mechanisms of IFN antagonism and their roles in disease. To assess P3-MT interactions, we used confocal laser scanning microscopy (CLSM) for a newly developed quantitative live-cell assay of protein-MT association, and super-resolution direct stochastic optical reconstruction microscopy (*d*STORM). The latter is a single-molecule localization technique that detects photon emissions from single, spatially and temporally distinct, fluorescent molecules to precisely localize individual fluorophores; this can achieve c. 20 nm spatial resolution to enable analysis of structural changes to single MT filaments (c. 25 nm diameter). Using these approaches together with quantitative colocalization analysis, yeast-2-hybrid assays, IFN-dependent reporter gene assays, and viral reverse genetics/animal infection, we show that MT-interaction and IFN/STAT1 antagonism differs significantly between P3 protein from the pathogenic and non-pathogenic viruses. We further identify a single residue mutation (N_226_-H) that significantly inhibits both processes, enabling the first direct analysis of the role of this interaction in viral pathogenicity in mice.

## Results and Discussion

### MT-interaction differs significantly between P3 from pathogenic and non-pathogenic RABV

**Figure 1:**
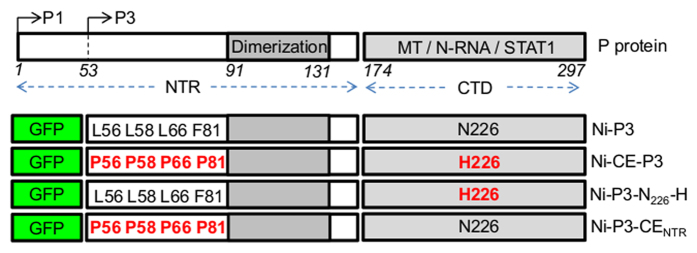
Schematic representation of P protein and the P3 proteins used in this study. P3 is an N-terminally truncated isoform of P protein comprising residues 53–297, which is generated in infected cells by ribosomal leaky scanning that results in translation from an internal AUG codon corresponding to M_53_ (arrow) of the full length P protein (called P1)^10^. The CTD (containing the MT, N-RNA and STAT1 binding regions) and NTR (containing the dimerization domain) are indicated; residue positions are indicated beneath the P1 protein. Residues at positions 56, 58, 66, 81 and 226 differ between P3 from the pathogenic (Ni) and attenuated (Ni-CE) strains of RABV (substitutions in Ni-CE-P3 are in red).

To assess the relationship of P3-MT-association with pathogenicity we expressed P3 from the Ni and Ni-CE RABV strains fused to green fluorescent protein (GFP; Fig. 1) in a panel of mammalian cell lines from neuronal and extraneural tissues of several RABV susceptible/host species^23^,^24^,^25^,^26^, including COS-7 (African green monkey kidney), NSC-34 (mouse motor neuron-like), SK-N-SH (human neuronal), HeLa (human epithelial) and NA (mouse neuroblastoma). Expression of the proteins was confirmed by Western analysis lysates of COS-7 cells (see supplemental Fig. S1).

**Figure 2:**
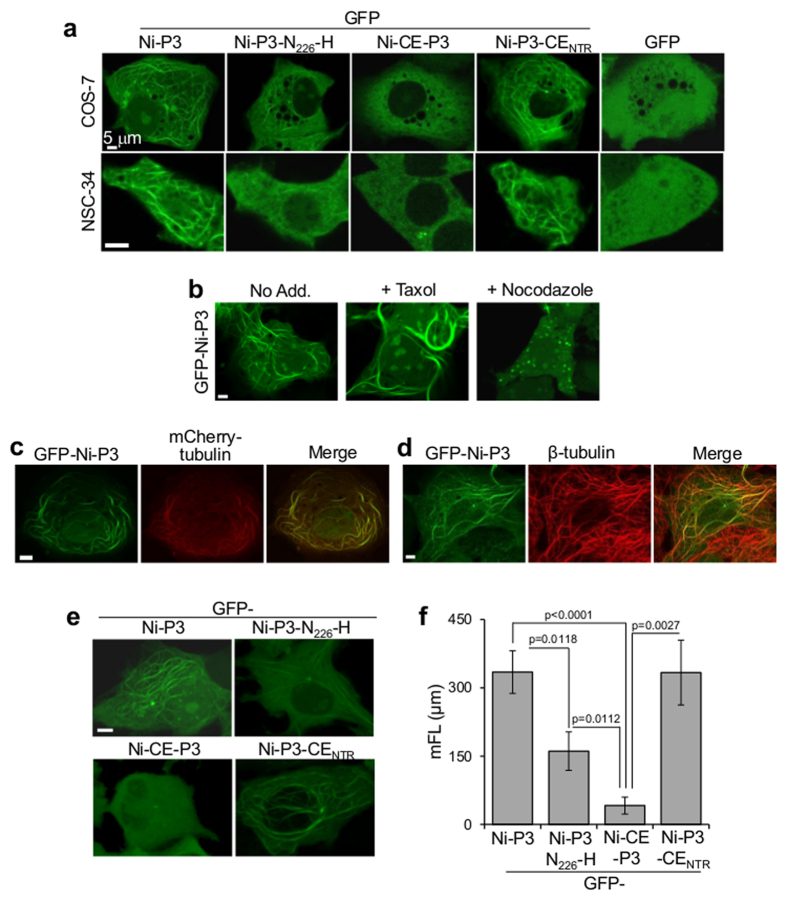
Interaction of Ni-P3 with MTs is impaired by N_226_-H mutation. (**a**) COS-7 cells were transfected to express the indicated proteins before analysis by live-cell CLSM; each image is representative of cells in 30 fields of view sampled over 3 separate assays (COS-7) or 9 fields of view (NSC-34). (**b–d**) COS-7 cells transfected to express GFP-Ni-P3 were treated with or without Taxol or nocodazole (**b**) co-transfected to express mCherry-tubulin (**c**) or fixed and immunostained for β-tubulin (**d**) before analysis by CLSM; colocalization in b and d is apparent as yellow coloration in merged image. (**e**) Live COS-7 cells expressing the indicated proteins were analyzed by CLSM to generate deconvoluted 3D images (images show reconstructed 3D images viewed down the z-axis). (**f**) Images such as those shown in (**e**) were analyzed to derive mean filament length values (mFL ± SEM; n ≥ 30 cells from 3 identical assays). p values were determined using the Mann Whitney test.

We previously found that MT interaction by GFP-fused P3 of the RABV CVSII strain can be detected in living cells by CLSM as association with a cytoplasmic filamentous network^19^. Consistent with this, extensive interaction of GFP-Ni-P3 with cytoplasmic filaments was observed in all cell types tested (COS-7, NSC-34, shown in Fig. 2a, and SK-N-SH, HeLa, NA, not shown). Importantly, GFP-Ni-CE-P3 filament association was clearly impaired in each of these cell types and comparable differences were observed between non-fused Ni-P3 and Ni-CE-P3 in fixed immunostained COS-7 cells (see supplementary Fig. S2). The Ni-P3-associated filaments were confirmed to be MTs based on sensitivity to MT-targeting drugs (Fig. 2b), and colocalization with mCherry-tubulin in living cells (Fig. 2c) and endogenous tubulin in immunostained cells (Fig. 2d). Thus, MT association differs between P3 proteins from pathogenic and attenuated virus.

The Ni-CE *P* gene contains mutations compared with Ni *P* that result in 5 amino acid residue substitutions, one or more of which account for the differing function in IFN-antagonism and pathogenicity^13^,^20^,^21^. All of the substitutions affect the P3 sequence (Fig. 1), resulting in four proline substitutions clustered within the N-terminal region (NTR), and one histidine substitution (N_226_-H) in the globular C-terminal domain (CTD). Previous mapping studies indicated that the latter domain mediates association with MTs^19^. To examine the effects of specific mutations, we expressed GFP-Ni-P3 containing N_226_-H alone (GFP-Ni-P3-N_226_-H) or the NTR mutations (GFP-Ni-P3-CE_NTR_) for analysis as above. MT filament association of GFP-Ni-P3-N_226_-H, but not GFP-Ni-P3-CE_NTR_, was clearly impaired in COS-7, NSC-34 (Fig. 2a), and SK-N-SH, HeLa, and NA cells (not shown).

To quantify the extent of MT network association, and effects thereon, of Ni-CE mutations we generated deconvoluted 3D images of living cells expressing GFP-fused P3 proteins (images viewed down the z-axis are shown in Fig. 2e), and developed a methodology to detect and measure intracellular filamentous GFP using the *Imaris* software filament-tracing algorithm tool (see Materials and Methods); this is to our knowledge the first application of this tool to quantify protein-MT interaction in living cells. This enabled semi-automated detection of P3-associated filaments in single cells to calculate the total filament length. The corresponding *mean filament length* (mFL) per cell was then determined for ≥ 30 cells for each protein tested (Fig. 2f). Results confirmed that MT-association of GFP-Ni-CE-P3 and GFP-Ni-P3-N_226_-H, but not GFP-Ni-P3-CE_NTR_, was significantly reduced compared to GFP-Ni-P3 (p < 0.0001 and p = 0.0118 for Ni-CE and Ni-P3-N_226_-H, respectively), with the N_226_-H mutation alone causing a c. 50% decrease in the mFL of Ni-P3. Thus, N_226_-H, but not the NTR mutations, directly impacts on P3-MT association. However, since N_226_-H alone does not fully recapitulate the phenotype of Ni-CE-P3 (Fig. 2f) it appears the NTR mutations can augment the effect of N_226_-H, indicative of an indirect/regulatory role of the NTR.

**Figure 3:**
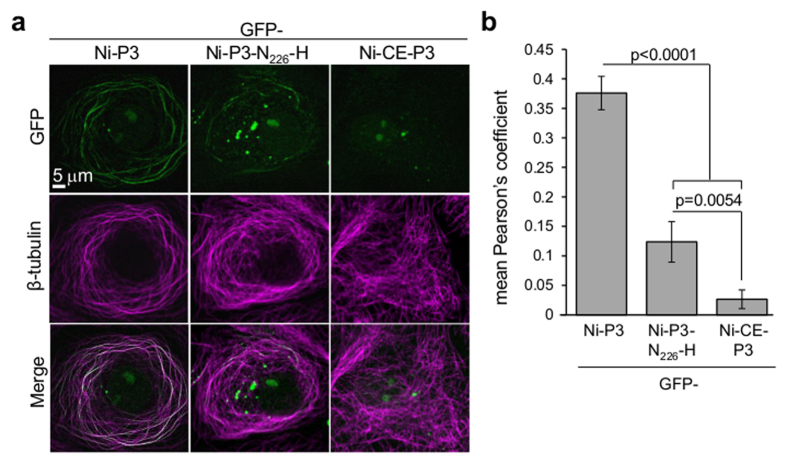
N_226_-H mutation inhibits colocalization of P3 with MTs (**a**) COS-7 cells expressing the indicated proteins were extracted to remove soluble (non-MT-associated) protein before fixation and immunostaining for β-tubulin, and analysis by CLSM (colocalization is apparent as white coloration in merged image). (**b**) Images such as those shown in (**a**) were analyzed to derive Pearson’s coefficient as a measure of colocalization between GFP-P3 and β-tubulin (mean ± SEM; n ≥ 14 cells from two assays). p values were determined using Student’s t-test or the Mann-Whitney test.

To confirm that the reduced mFL for Ni-CE-P3 and Ni-P3-N_226_-H is due to decreased interaction with MTs, we performed cytoplasmic extraction of transfected cells to remove non-MT associated protein, as described^27^, before immunostaining for MTs and CLSM analysis. Quantitation of P3-MT colocalization using Pearson’s coefficient indicated that MT-association is significantly (p < 0.0001) impaired for GFP-Ni-CE-P3 and GFP-Ni-P3-N_226_-H compared with GFP-Ni-P3 (Fig. 3). However, the capacity of Ni-P3-N_226_-H to associate with MTs remained greater than that of Ni-CE-P3, consistent with data from the live-cell filament tracing assays (Fig. 2f).

### MT bundling by P3 is significantly impaired by N_226_-H mutation

Interactions of cellular and viral MAPs with MTs often induce gross structural changes in MT networks that can be detected by light microscopy/CLSM analysis and are thought to correspond to MT bundling^28^,^29^,^30^,^31^,^32^,^33^,^34^,^35^. Such effects could provide a means to quantify MAP-MT interactions at the level of individual MT filaments. However, light microscopy/CLSM is diffraction limited with the greatest possible spatial resolution c. 200 nm, precluding direct quantitative analysis of individual MT filaments (c. 25 nm diameter) or of the extent of filament bundling. While electron microscopy (EM) can visualize individual filaments, and has been used to confirm bundling activity of certain MAPs^28^,^29^,^30^,^31^,^32^,^33^,^34^,^35^, such studies are typically limited to complexes of purified protein with *in vitro* polymerized tubulin, or analysis of cells subjected to extensive chemical fixation procedures/sample preparation times^36^,^37^. Furthermore, EM precludes the use of fluorescent tags for precise localization of structures/proteins of interest in large cell populations. Thus, to directly detect and quantify cellular MT filament bundling induced by P3, we utilized *d*STORM super-resolution imaging, which overcomes the diffraction limit by detecting unperturbed emission point spread functions from single molecules, enabling highly precise spatial localization of the emitting fluorophores. Analysis used cell fixation and immunostaining protocols known to preserve MT integrity, which we have been used previously to visualize individual MTs^38^,^39^,^40^, with *d*STORM measurements made using a custom-built super-resolution widefield microscope that can achieve single molecule localization precisions better than 10 nm, and spatial resolution down to 20 nm^41^. Detailed images of MT architecture were acquired with sufficient resolution to differentiate closely associated MTs (presumed to be bundles, see below) from individual MTs which are tens of nanometers apart but occupy the same diffraction limited area and so cannot be resolved using conventional light microscopy (see supplementary Fig. S3).

**Figure 4:**
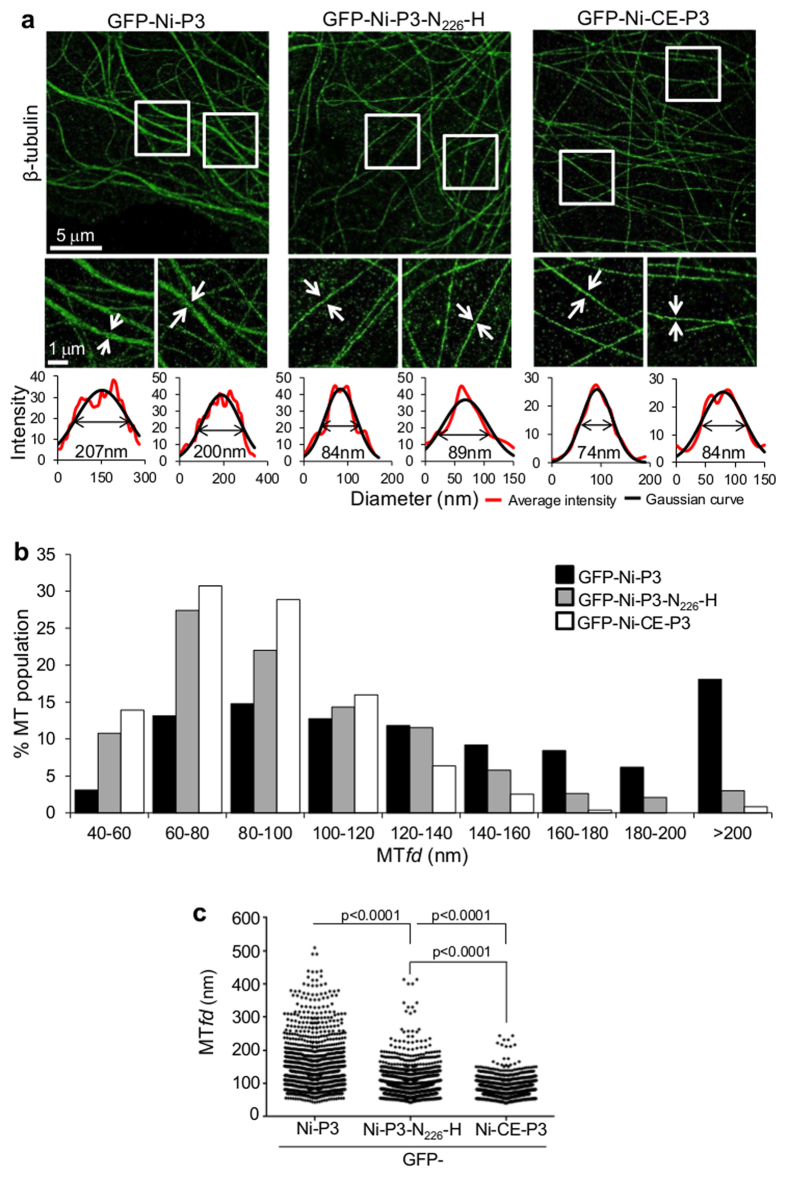
Quantitative *d*STORM analysis of P3-induced bundling of MTs. (**a**) *d*STORM images of immunostained β**-**tubulin in COS-7 cells expressing the indicated proteins are shown in the upper panels. Boxed areas are expanded in the lower panels, and Gaussian functions (black line) fit to the average intensity profile (red line) of the indicated filaments shown below. The derived MT*fd* (calculated at the full width half-height of the Gaussian function) is indicated. (**b**) The frequency distribution of MT*fd*s calculated for each protein is shown (n = 1294 [GFP-Ni-P3], 1071 [GFP-Ni-CE-P3] and 1299 [GFP-Ni-P3-N_226_-H]; measurements are from 10 cells for each protein over two identical assays). (**c**) Scatter dot plots of MT*fd*s shown in (**b**). p values were determined using the Mann-Whitney test.

To quantitatively analyze the dimensions of the filaments and occurrence of bundling in *d*STORM images, we calculated the relative MT *feature diameter* (MT*fd*) using the width at half-height of a Gaussian function fit to the average intensity profile of a cross-section of a continuous MT feature (Fig. 4a). The width of single MT filaments imaged by this method will be greater than the intrinsic diameter of a MT filament (c. 25 nm) since it is the locations of antibody-conjugated Alexa Fluor-647 molecules that are detected. Thus, the primary immunolabel against tubulin and the Alex Fluor 647-conjugated secondary immunolabel (which label both sides of the filament) add some 30–40 nm to the apparent width of a single MT. Taking this and the localization precision of the fluorophores themselves (typically 9–15 nm in these measurements) into account, we predicted a range of 40–100 nm for single MTs. In agreement with this, benchmarking using mock-transfected cells indicated that 72% of MT*fd*s were in this range (see supplementary Fig. S3). The thicker filaments detected in mock transfected cells (>100 nm) are likely the result of native bundling of MTs as well as detection of overlapping MTs that are separated in axial space but appear to associate in the 2D *d*STORM image. Nevertheless, the majority of features are within the range expected for a population of largely individual, non-bundled MTs.

Analysis of GFP-Ni-P3-expressing cells (Fig. 4a) indicated a substantial change in MT architecture, with 63.4% of MT*fd*s exceeding 40–100 nm, and 18.1% exceeding 200 nm (Fig. 4b), indicative of extensive bundling. The notion that these large MT features correspond to multiplex bundles of single MT filaments is supported by the observation that the thicker filaments often appeared to split into thinner filaments, with the continuous length of the “bundled” region indicative of specific association of the constituent MTs (see supplementary Fig. S4).

The architecture of MT networks in cells expressing GFP-Ni-CE-P3 and GFP-Ni-P3-N_226_-H (Fig. 4a) was much more consistent with that observed in mock-transfected cells than in cells expressing GFP-Ni-P3, with 78.5% (GFP-Ni-CE-P3) and 67.3% (GFP-Ni-P3-N_226_-H) of MT*fd*s within 40–100 nm (Fig. 4b), indicative of defective bundling by these proteins. However, Ni-P3-N_226_-H retained a slightly increased bundling capacity compared to Ni-CE-P3, indicated by a > 2 fold higher proportion of MT*fd*s in all bins of the frequency distribution exceeding 140 nm. Consistent with the observed trend, statistical analysis indicated that the median MT*fd* for cells expressing GFP-Ni-P3 was significantly (p < 0.0001) greater than that for GFP-Ni-P3-N_226_-H, which in turn was significantly (p < 0.0001) greater than that for GFP-Ni-CE-P3 (Fig. 4c).

Thus, it appears that N_226_-H substantially inhibits bundling, but does not fully recapitulate the defective phenotype of Ni-CE-P3, so that the degree of bundling detected by *d*STORM correlates with the extent of MT network association determined in CLSM-based filament tracing and co-localization assays (Figs 2 and 3). These data indicate that *d*STORM provides an effective method to quantify protein-MT association at the level of individual filaments. This is to our knowledge the first application of *d*STORM to interrogate viral protein-MT interaction and highlights the power of *d*STORM to elucidate fundamental processes at the virus-host interface. Together, the data indicate that N_226_-H directly impacts on P3-MT-association, consistent with its localization to the CTD that contains MT-association activity (Fig. 1)^19^.

### Interaction of P3 with RABV N-protein and homodimerisation of P protein is not impaired by mutations in Ni-CE

**Figure 5:**
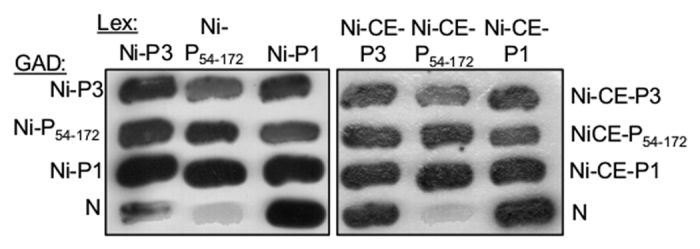
Ni-CE P can interact with N-protein and contains a functional homodimerisation region. L40 yeast cells were cotransformed to express the DNA-binding domain of LexA (Lex) or the GAL4-activation domain (GAD) fused to Ni-P1, Ni-CE-P1, Ni-P3, Ni-CE P3, the corresponding P3 NTRs (P_54-172_), or RABV N protein. Growth streaks are shown, where interaction of Lex- and GAD-fused proteins is indicated by dark coloration of colonies.

P protein has critical roles in viral genome replication, which requires interaction of the CTD with RABV nucleoprotein (N protein) to mediate association of P protein with N-protein-encapsidated genomic RNA (N-RNA; Fig. 1)^42^. The CTD also mediates interaction with STAT1 (Fig. 1), which is important to the IFN-antagonistic functions of P protein isoforms^15^,^16^,^43^,^44^. The reported N- and STAT1-binding sites are distinct from N_226_^44^,^45^,^46^, and previous data indicate that they are not significantly impacted by N_226_-H, as Ni-CE virus is highly viable in cultured cells, and Ni-CE-P is not deficient for STAT1 interaction^13^. To confirm directly that Ni-CE-P CTD interaction with N-protein is unimpaired, we used yeast-2-hybrid analysis (Fig. 5), which has been used extensively to identify and characterize bi-molecular interactions of P protein, including with N protein, as well as P protein homodimerisation^47^,^48^,^49^. This indicated no evident defect for Ni-CE compared with Ni P proteins. 2-hybrid analysis of homodimerization of P protein via the NTR-localized dimerization domain^50^ (Fig. 1), which has been shown to be required for P3-MT association via the CTD and for MT-dependent inhibition of STAT1^19^, also indicated no defect in Ni-CE-P (Fig. 5). Thus, the mutations in Ni-CE do not appear to be generally detrimental to P protein structure or function, indicative of specific effects on MT interaction such that the N_226_-H mutation could be applied to investigate directly the functional significance of P3-MT association.

### N_226_-H mutation impairs IFN antagonist function of P3 and pathogenicity of RABV

**Figure 6:**
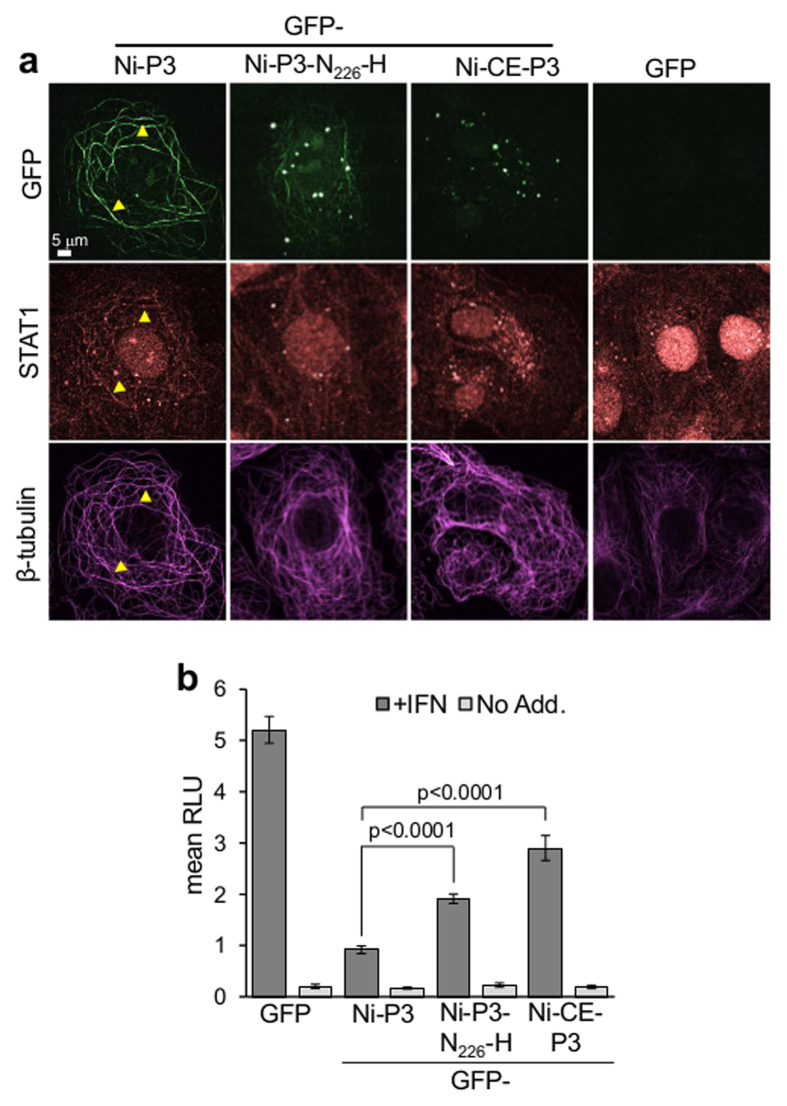
N_226_-H mutation impairs the IFN-antagonistic function of P3. (**a**) IFNα-treated COS-7 cells expressing the indicated proteins were extracted before fixation and immunostaining for STAT1 and β-tubulin, and analysis by CLSM; images are representative of 15 fields of view from two assays. (**b**) IFN-α-dependent signaling in COS-7 cells expressing the indicated proteins was analyzed using a dual luciferase reporter gene assay, as previously described^13^,^19^,^43^. Luciferase activity is expressed as fold change relative to that obtained for IFN-α-treated cells expressing Ni-P3 protein (mean relative light units [RLU] ± SEM; n = 12 from 4 identical assays). p values were calculated using Student’s t-test.

To examine the effect of N_226_-H mutation on the capacity of P3 to cause association of STAT1 with MTs, cells expressing GFP-fused Ni-P3, Ni-CE-P3 and Ni-P3-N_226_-H were treated with IFNα before cytoplasmic extraction, fixation and immunostaining for STAT1 and tubulin, and imaging by CLSM. Association of GFP-Ni-P3 and STAT1 with MTs was clearly detectable (Fig. 6a), but association of STAT1 with MTs was strongly reduced or undetectable in cells expressing GFP-Ni-P3-N_226_-H, and there was no evidence of STAT1-MT interaction in cells expressing GFP-Ni-CE-P3 or control cells transfected to express GFP alone. To further analyze the effect of N_226_-H on P3-mediated antagonism of IFNα/STAT1-dependent signaling, we used a luciferase reporter gene assay, as previously described^13^,^19^,^44^. Ni-P3 strongly inhibited signaling in response to IFNα, but Ni-CE-P3 and Ni-P3-N_226_-H were significantly (p < 0.0001) impaired in this respect (Fig. 6b). Notably, the IFN-antagonistic function of the different P3 proteins correlated with their capacity to associate with MTs and induce MT-association of STAT1 (Figs 2, 3, 4 and 6a), and with the pathogenicity of Ni and Ni-CE viruses.

**Figure 7:**
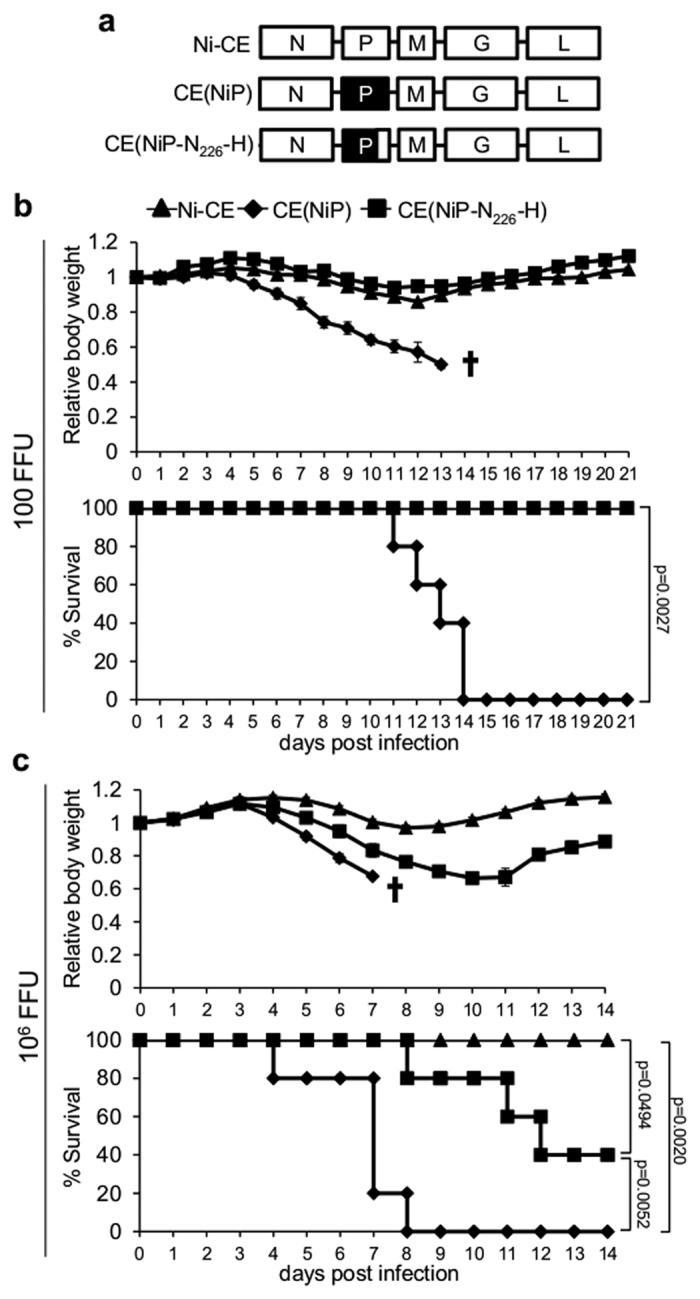
N_226_-H mutation attenuates RABV. (**a**) Schematic representation of the genomes of viruses used; genes from Ni and Ni-CE are in black and white, respectively. The *P* gene of Ni-CE is substituted for the *P* gene of Ni in CE(NiP), and for a mutated version of the Ni *P* gene containing N_226_-H in CE(NiP-N_226_-H). (**b,c**) 100 (**b**) or 10^6^ (**c**) FFU of the indicated virus was inoculated intracerebrally into mice (five mice per condition) and body weight relative to that at 0 dpi (mean relative body weight ± SEM, for live mice) and survival was monitored over 21 or 14 dpi. p-values for survival curves were calculated using log-rank (Mantel-Cox) test. ^†^all mice dead or sacrificed on reaching end point.

To directly investigate the effect of N_226_-H on infection *in vivo*, we introduced this mutation alone into the Ni *P* gene of the pathogenic IFN-resistant CE(NiP) virus^13^,^21^; the derived recombinant virus was called CE(NiP-N_226_-H) (Fig. 7a). ddY mice (five mice per virus) were inoculated intracerebrally with 100 focus forming units (FFU) of Ni-CE, CE(NiP) or CE(NiP-N_226_-H) virus, and monitored over 21 days post-infection (dpi), as previously^13^. Consistent with previous observations, mock infection was asymptomatic (data not shown), with infection by Ni-CE virus causing only mild and temporary weight changes, while CE(NiP) caused death or sacrifice at the defined end-point in 1 out of 5 mice by 11 dpi, and 5/5 mice by 14 dpi (Fig. 7b). The outcome of infection with CE(NiP-N_226_-H) was similar to infection with Ni-CE, causing only minor temporary weight loss with no onset of major symptoms and no fatalities, indicating significant attenuation (p = 0.0027). We also performed infection using 10^6^ FFU virus, finding that infection by Ni-CE remained non-lethal while all CE(NiP) infected mice succumbed to infection by 8 dpi. Significant (p = 0.0052) attenuation of CE(NiP-N_226_-H) was observed an the increased FFU, with only 3/5 mice succumbing by 14 dpi while the remaining mice showed increasing weight from 11 dpi.

Taken together, our data indicate that N_226_-H mutation impairs the capacity of P3 to interact with MTs and effect antagonism of antiviral signaling, and strongly reduces the ability of RABV to cause lethal infection in mice. These findings are consistent with key roles for MT-dependent IFN-antagonism through the P3-MT interface in the pathogenicity of RABV, supporting the hypothesis that viral protein-MT interactions have significant roles in subversion of host biology distinct from well-established functions of MTs in virus trafficking^9^. Importantly, the observation that modification of these interactions can affect disease outcomes *in vivo* identifies new potential targets at the virus-MT interface that could contribute to the development of attenuated vaccines and/or antiviral drugs to combat infection by lyssaviruses, which cause rabies disease with an almost 100% case-fatality rate and no effective therapeutic options^51^.

## Materials and Methods

### Ethics statement

Animal experiments were conducted in strict accordance with the *Regulations for Animal Experiments* in Gifu University and the *Standards Relating to the Care and Management of Experimental Animals* (Notice No. 6 of the Prime Minister’s office, March 27, 1980), based on the *Law for the Humane Treatment and Management of Animals* (Ministry of the Environment, Japan). The protocols were approved by the *Committee for Animal Research and Welfare* of Gifu University (Approval Number 08119).

### Constructs

Constructs were generated by PCR amplification of inserts from Ni and Ni-CE P gene cDNA^13^ for cloning into the mammalian expression vector pEGFP-C1 (to express GFP-fused protein) or pΔEGFPC1 (to express untagged protein), as previously^52^,^53^. Ni-P3-N_226_-H and Ni-P3-CE_NTR_ cDNA was generated by PCR overlap mutagenesis. For yeast-2-hybrid analysis, cDNA encoding RABV P or N protein sequences were cloned into pLex or pGAD (Clontech) plasmids. The mCherry-tubulin plasmid was a kind gift of R. Tsien (University of California)^54^. Luciferase assays used the plasmids pRL-tk (encoding *Renilla* luciferase under the control of a constitutively active promoter) and pISRE-luc (encoding *Firefly* luciferase under the control of an IFNα inducible promoter)^43^,^44^,^55^. All constructs were verified by Sanger sequencing.

### Cell culture, transfection and drug treatments

COS-7 and NSC-34 cells were cultured in DMEM supplemented with 10% FCS (37 °C, 5% CO_2_). Cells were grown to 80% confluency in 6-well culture dishes (for luciferase assays), on coverslips in 6-well culture dishes (for live-cell CLSM) or 12-well culture dishes (for fixed CLSM), or in 8-well Lab-Tek chambered coverglass (Nunc; for *d*STORM), before transfection using Lipofectamine 2000 or FugeneHD according to the manufacturer’s instructions. To disrupt or stabilize MTs, cells were treated with nocodazole (Sigma; 4 h, 5 μg/ml) or Taxol (Sigma; 4 h, 1 μg/ml), respectively^19^.

### Confocal laser scanning microscopy

For live cell analysis, cells were imaged in phenol free DMEM in a 37 °C heated chamber. For immunostaining of untagged P protein, cells were fixed with 100% methanol (10 mins, −20 °C) and immunostained using anti-P protein antibody^56^ and Alexa Fluor-568-conjugated secondary antibody (Molecular Probes) before mounting in Gold antifade mountant (Life Technologies). For immunostaining of MTs and STAT1, cells were treated with or without 1000 U/ml recombinant human IFNα (PBL Interferon Source) for 30 minutes before fixation with 3% paraformaldehyde/0.1% glutaraldehyde (10 minutes, 37 °C) and staining with anti-STAT1 (Santa Cruz) and/or anti-β-tubulin (Sigma), followed by Alexa Fluor-647 or Alexa Fluor-568-conjugated secondary antibody (Molecular Probes). To extract soluble (non-MT associated) protein, we used a modified version of the protocol of Zhai *et al*. 1996^27^ whereby cells were incubated in MT-stabilizing buffer (60 mM PIPES, 25 mM HEPES, 10 mM EGTA, 2 mM MgCl_2_, pH 6.9) containing 0.5% Triton-X100 (3 minutes, 37 °C) prior to fixation as above.

CLSM analysis used a Nikon Eclipse C1, Leica SP5 or Olympus fluorview FV1000 inverted confocal laser scanning microscope with 60x, 63x or 100x oil-immersion objective. Image acquisition used Nikon NIS-Elements (Eclipse C1), Leica LAS AF (SP5) or Olympus fluoview FV10-ASW (FV1000) software. Digitized confocal files (single slices) were processed using ImageJ 1.62 software (NIH). For colocalization analysis, Pearson’s coefficient was calculated using Velocity software (PerkinElmer).

### 3D live-cell imaging and calculation of mean filament length (mFL)

To generate deconvoluted 3D images of live cells, the point spread function of a Nikon Eclipse C1 inverted confocal laser scanning microscope with 60 × 1.4 NA oil immersion lens was calculated using 0.2 μm green fluorescent beads (Invitrogen). Images of beads were sampled along the z-axis at 0.25 μm intervals, and a 3D image of a single bead reconstructed from the z-stack was subjected to deconvolution using AutoQuant X software (MediaCybernetics; 20 iterations, high noise filtering). Live COS-7 cells expressing GFP-Ni-P3 or derivatives thereof were then imaged using identical conditions to generate 3D imaged from z-stacks, and these were deconvoluted using the determined point spread function.

For filament tracing, 3D images were analyzed using the Imaris (Bitplane) software. A typical image of a GFP-Ni-P3-expressing cell was initially analyzed to determine standard settings to detect filaments based on the difference in contrast between filamentous fluorescence and diffuse cytoplasmic fluorescence using the *filament tracing tool algorithm*. The settings were subsequently used for the analysis of all cells (n ≥ 30 cells for each construct tested). The total length of GFP-associated filaments for individual cells was calculated using the Imaris *filament length (sum)* value, and the mean value for n ≥ 30 cells (mFL) was calculated for each protein. Cells without traceable filaments were given a mFL of zero.

### Dual luciferase reporter assays

COS-7 cells were transfected with pRL-TK and pISRE-luc plasmids, and with plasmid encoding GFP-fused Ni-P3, Ni-CE-P3, Ni-P3-N_226_H or GFP alone, as previously^13^,^19^,^55^. Cells were treated 21 h post-transfection with or without 500 U/ml recombinant human IFNα (PBL Interferon Source) for 6 h, before measurement of luciferase activity according to the manufacturer’s protocol (Dual-Luciferase Reporter Assay System; Promega). Normalized luciferase activity was calculated by dividing Firefly luciferase values by Renilla luciferase values, before determination of the fold change relative to values obtained for IFNα-treated cells expressing wild-type Ni-P3 protein.

### *d*STORM microscopy

Cells were fixed 18 h post-transfection using 3% paraformaldehyde/0.1% glutaraldehyde (37 °C, 10 min), before permeabilization using 1% Triton-X100 (5 min) and blocking with 1% bovine serum albumin in phosphate buffered saline (PBS; 1 hr). Cells were then immunostained using anti-β-tubulin antibody (Sigma) and Alexa Fluor-647-conjugated secondary antibody (Molecular Probes), followed by a second fixation with 3% paraformaldehyde/0.1% glutaraldehyde (10 min). Fixed cells were imaged in a buffer containing 10% glucose, 120 mM mercaptoethylamine, 200 μg/mL glucose oxidase, and 20 μg/mL catalase in isotonic PBS (pH 8).

Cells were viewed using a custom-built widefield fluorescence microscope^41^ (Olympus IX71, 100 × 1.49 NA TIRF objective) with epifluorescence used to select GFP-positive cells (488 nm excitation). Excitation with a high power red laser (Oxxius Laser Boxx, 638 nm, ~3–5 kW cm^2^) was used to induce photoswitching of the Alexa Fluor-647 molecules. Once satisfactory photoswitching had been achieved, cells were imaged at 100 Hz over several minutes using an electron multiplying CCD camera (Andor Ixon Ultra) to collect a TIFF stack of 5,000–20,000 consecutive images containing single molecule emissions. Raw TIFF stacks were imported into rapi*d*STORM^57^ and analyzed using an input pixel size of 10 nm and a point spread function full width half height diameter of 370 nm, as determined using the ‘estimate PSF form’. Localizations of emissions from single molecules were only calculated when more than 500 photons were detected from a single location within a frame. These point spread functions were fit using a 2D Gaussian function and the resulting list of co-ordinates rendered into images using 10 nm pixels. Localization precisions were estimated using the Thompson equation^58^ while spatial resolution is quoted as ‘as good as’ figures based on cross-sections of features within images discernible by separation at half height.

To quantitate the occurrence of MT-bundling, a 10 × 10 grid was placed over a 25 μm^2^ region for each cell image and the average intensity profile was taken longitudinally across all filaments within each grid box. Gaussian functions were then fit to each average intensity profile and the MT *feature diameter* (MT*fd*) derived from the full width half-height.

### Yeast two-hybrid assays

Yeast L40 cells were co-transformed with pLex and pGAD constructs containing DNA encoding the indicated Lex- or GAD-fused proteins for an X-Gal (5-bromo-4-chloro-3-indolyl-β-D-galactosidase) overlay assay. Briefly, an X-Gal mixture containing 0.5% agar, 0.1% SDS, 6% dimethylformamide and 0.04% X-Gal was overlaid on fresh transformants grown on medium lacking tryptophan and leucine, and the appearance of growth streaks assessed up to 18 h at 30 °C; blue coloration (due to β-galactosidase activity in the X-Gal-containing medium) is indicative of interaction of Lex- and GAD-fused proteins.

### Generation of recombinant virus

Ni-CE and CE(NiP) viruses have been described previously^20^. Full-genome plasmid for CE(NiP-N_226_-H) was constructed using Ni-CE strain plasmid by conventional molecular cloning techniques (detailed information is available on request) and the mutant virus was recovered as described previously^59^. P gene sequences were confirmed by sequencing of RT-PCR fragments from the recombinant viruses, and viral stocks were propagated in mouse neuroblastoma NA cells.

### Pathogenicity studies

Five 4-week-old female ddY mice (Japan SLC Inc., Hamamatsu, Japan) were intracerebrally inoculated with 0.03 ml dilutent (E-MEM with 5% FCS) containing 100 FFU virus, 10^6^ FFU of virus or no virus (mock infection). The weight of individual mice was measured daily over 14 or 21 dpi and body weight change calculated relative to the weight at 0 dpi. Mice failing to show righting reflexes in body tilt experiments (end-point) were sacrificed.

### Statistical analysis

Prism version 6 software (Graphpad) was used for statistical analysis to calculate p-values using Student’s t-test (unpaired, two-tailed). If variances were determined to be significantly different (F-test) a Welch’s correction was applied. If datasets failed the D’Agostino-Pearson omnibus normality test, the alternative Mann-Whitney test was used. To calculate p-values for survival curves the log-rank (Mantel-Cox) test was used.

## Additional Information

**How to cite this article**: Brice, A. *et al*. Quantitative Analysis of the Microtubule Interaction of Rabies Virus P3 Protein: Roles in Immune Evasion and Pathogenesis. *Sci. Rep.* **6**, 33493; doi: 10.1038/srep33493 (2016).

## Supplementary Material

Supplementary Information
