# Supplementary material for: Quantitative Analysis of the Microtubule Interaction of Rabies Virus P3 Protein: Roles in Immune Evasion and Pathogenesis
Source: Sci Rep. 2016 Sep 21;6:33493. doi: 10.1038/srep33493 (PMC5030706; doi:10.1038/srep33493)
Supplement: Supplementary Information [file srep33493-s1.pdf]

QUANTITATIVE ANALYSIS OF THE MICROTUBULE INTERACTION OF RABIES VIRUS P3 PROTEIN: ROLES IN IMMUNE EVASION AND PATHOGENESIS

Aaron Brice[1], Donna R. Whelan[2], Naoto Ito[3,4], Kenta Shimizu[4], Linda Wiltzer-Bach[5,6], Camden Y. Lo[7], Danielle Blondel[8], David A. Jans[5], Toby D. M. Bell[2#], Gregory W. Moseley[1#]

[1]Viral Pathogenesis Laboratory, Department of Biochemistry and Molecular Biology, Bio21 Institute, The University of Melbourne, Melbourne, Victoria, 3010; [2]School of Chemistry, Monash University, Clayton, Victoria 3800, Australia; [3]Laboratory of Zoonotic Diseases, Faculty of Applied Biological Sciences, and [4]the United Graduate School of Veterinary Sciences, Gifu University, 1-1 Yanagido, Gifu 501-1193, Japan; [5]Nuclear Signaling Laboratory, Department of Biochemistry and Molecular Biology, Monash University, Clayton, Victoria 3800, Australia; [6]Current Address: Institute of Medical Virology, University Clinic Tuebingen, Elfriede-Aulhorn-Straße 6, 72076 Tübingen, Germany; [7]Monash Micro Imaging, 27-31 Wright Street, Clayton, Victoria, Australia, 3168; [8]Unité de Virologie Moleculaire et Structurale, CNRS, UPR 3296, 91198 Gif sur Yvette Cedex, France.

[#]**Corresponding Authors:** Dr. Gregory W. Moseley, Dept. of Biochemistry and Molecular Biology, Bio21 Institute, The University of Melbourne, Victoria 3010, Australia. Phone: 61-3-83442288, Fax: 61-3- 93481421. E-mail: gregory.moseley@unimelb.edu.au

Dr. Toby D.M. Bell, School of Chemistry, Monash University, Wellington Road, Clayton, Victoria 3800, Australia. Phone: 61-3-99054566, Fax: 61-3-99054597. E-mail: toby.bell@monash.edu

Supplementary Figures

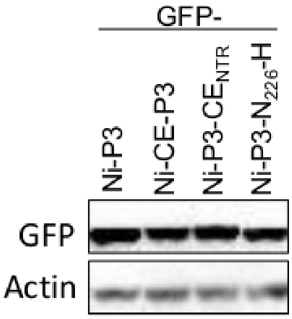

Figure S1. Western analysis of GFP-P3 protein expression in COS-7 cells Lysates of COS-7 cells expressing the indicated proteins were analyzed by Western blotting using anti-GFP and anti-actin antibody.

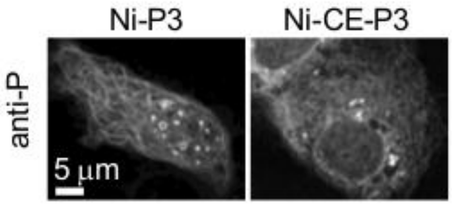

Figure S2. Interaction of non-fused Ni-CE-P3 and Ni-P3 protein with MTs COS-7 cells expressing non-fused Ni-P3 or Ni-CE-P3 were fixed and immunostained using anti-RABV P protein antibody[1] and Alexa-Fluor-568 conjugated secondary antibody before analysis by CLSM.

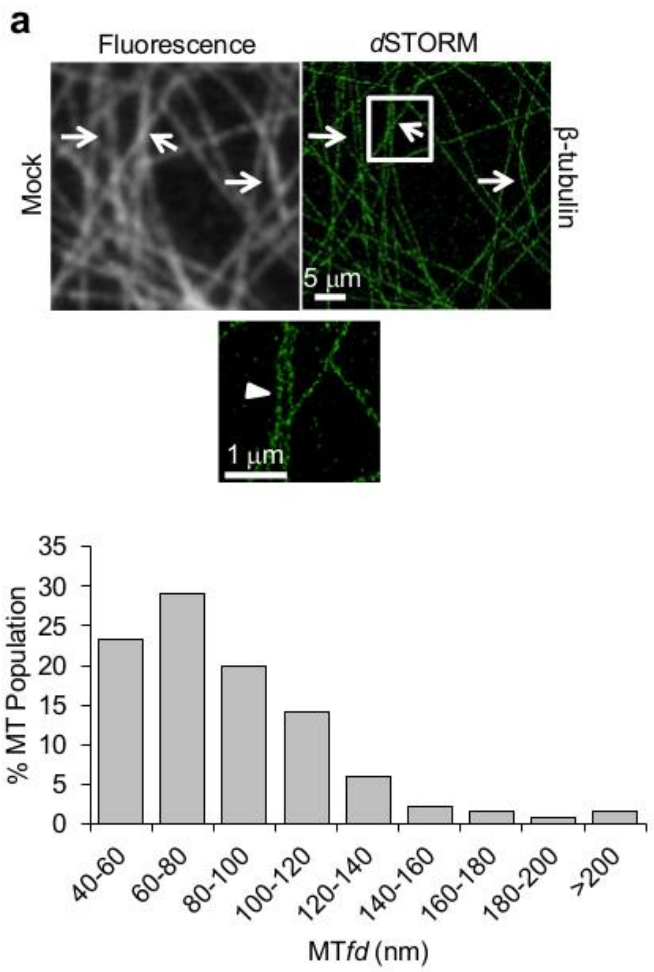

Figure S3. Benchmarking MT*fd* values from *d*STORM analysis of mock transfected cells (a) Conventional fluorescence image (upper left panel) and corresponding *d*STORM analysis (upper right panel) of a mock transfected COS-7 cell fixed and immunostained for β-tubulin; the boxed region of the *d*STORM image is expanded in the lower panel. Arrows indicate diffraction-limited proximally localized MTs that are well resolved in the *d*STORM analysis but appear as single filaments in the conventional fluorescence image. (b) The distribution of MT*fd*s measured in *d*STORM images such as those in (a) is shown (n = 1081 measurements from 10 cells sampled over 2 assays).

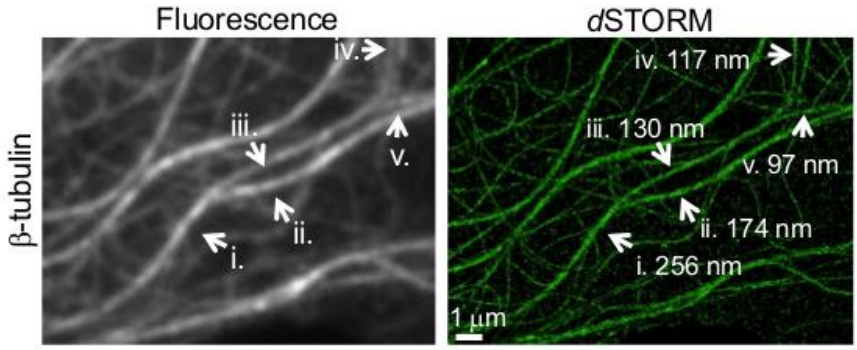

Figure S4. Example of a bundled MT feature. *d*STORM analysis of immunostained tubulin in a COS-7 cell expressing GFP-Ni-P3 is shown (left panel shows conventional fluorescence image, right panel shows *d*STORM image with derived MT*fd* measurements indicated). A single thick MT filament feature (i., MT*fd* = 256 nm) is observed to split into two separate filaments (ii. and iii., MT*fd*s of 174 nm and 130 nm). Filament iii. subsequently splits into two further filaments (iv. and v. MT*fd*s of 117 nm and 97 nm).

References

1 Lahaye, X. *et al.* Functional characterization of Negri bodies (NBs) in rabies virus-infected cells: Evidence that NBs are sites of viral transcription and replication. *Journal of virology* **83**, 7948-7958, doi:10.1128/JVI.00554-09 (2009).
